# Comparison of consistency in centiloid scale among different analytical methods in amyloid PET: the CapAIBL, VIZCalc, and Amyquant methods

**DOI:** 10.1007/s12149-024-01919-3

**Published:** 2024-03-21

**Authors:** Cong Shang, Keita Sakurai, Takashi Nihashi, Yutaka Arahata, Akinori Takeda, Kazunari Ishii, Kenji Ishii, Hiroshi Matsuda, Kengo Ito, Takashi Kato, Hiroshi Toyama, Akinori Nakamura, BATON Study Group

**Affiliations:** 1 Department of Radiology, National Center for Geriatrics and Gerontology, 7-430 Morioka-Cho, Obu, Aichi 474-8511 Japan; 2 Department of Radiology, Fujita Health University School of Medicine, Toyoake, Japan; 3 Department of Neurology, National Center for Geriatrics and Gerontology, Obu, Japan; 4 Department of Radiology, Faculty of Medicine, Kindai University, Osakasayama, Japan; 5 Team for Neuroimaging Research, Tokyo Metropolitan Institute of Gerontology, Tokyo, Japan; 6 Department of Biofunctional Imaging, Fukushima Medical University, Fukushima, Japan; 7 Drug Discovery and Cyclotron Research Center, Southern Tohoku Research Institute for Neuroscience, Koriyama, Japan; 8 Department of Clinical and Experimental Neuroimaging, National Center for Geriatrics and Gerontology, Obu, Japan; 9 Department of Biomarker Research, National Center for Geriatrics and Gerontology, Obu, Japan

**Keywords:** Amyloid PET, Amyquant, CapAIBL, Centiloid scale, VIZCalc, Positron emission tomography

## Abstract

**Objective:**

The Centiloid (CL) scale is a standardized measure for quantifying amyloid deposition in amyloid positron emission tomography (PET) imaging. We aimed to assess the agreement among 3 CL calculation methods: CapAIBL, VIZCalc, and Amyquant.

**Methods:**

This study included 192 participants (mean age: 71.5 years, range: 50–87 years), comprising 55 with Alzheimer’s disease, 65 with mild cognitive impairment, 13 with non-Alzheimer's dementia, and 59 cognitively normal participants. All the participants were assessed using the three CL calculation methods. Spearman’s rank correlation, linear regression, Friedman tests, Wilcoxon signed-rank tests, and Bland–Altman analysis were employed to assess data correlations, linear associations, method differences, and systematic bias, respectively.

**Results:**

Strong correlations (rho = 0.99, *p* < .001) were observed among the CL values calculated using the three methods. Scatter plots and regression lines visually confirmed these strong correlations and met the validation criteria. Despite the robust correlations, a significant difference in CL value between CapAIBL and Amyquant was observed (36.1 ± 39.7 vs. 34.9 ± 39.4; *p* < .001). In contrast, no significant differences were found between CapAIBL and VIZCalc or between VIZCalc and Amyquant. The Bland–Altman analysis showed no observable systematic bias between the methods.

**Conclusions:**

The study demonstrated strong agreement among the three methods for calculating CL values. Despite minor variations in the absolute values of the Centiloid scores obtained using these methods, the overall agreement suggests that they are interchangeable.

## Introduction

Quantitative analysis in amyloid positron emission tomography (PET) is expected to allow a continuous measure of amyloid burden, which can be used to complement dichotomous visual reads. Quantification may facilitate comparisons between different tracers and allow for more reliable assessments of amyloid burden across diverse settings [1]. Although the findings from clinical trials are controversial, quantitative analysis is expected to detect early amyloid abnormalities in cognitively unimpaired individuals, helping identify the optimal window for treatment intervention [2]. Moreover, quantification serves as a reference marker and facilitates the monitoring of changes in amyloid deposition over time [3]. When used as an adjunct to visual interpretation, quantitative analysis enhances readers’ performance and confidence when interpreting scans [4]. Furthermore, inter-reader agreement in the interpretation of PET scans is improved, thereby effectively reducing the variability in results [5].

The Centiloid (CL) scale, initially introduced by Klunk et al. [1], is a standardized transformation of semi-quantitative amyloid imaging data. CL is designed to convert global cortical 50–70 min PiB PET standardized uptake value ratio (SUVR) data to a scale anchored at 0, representing relatively "high certainty" amyloid-negative individuals, and at 100, representing individuals with definitive amyloid deposition in the cortex.

In recent years, substantial progress has been made in calculating CL values and/or SUVR from amyloid PET images. Some of these methods use computed tomography (CT) instead of magnetic resonance imaging (MRI) for anatomical reference because they are more readily available [6, 7]. Additionally, some methods do not require MRI or CT anatomical assistance, allowing automated analysis of amyloid PET images while optimizing accurate spatial normalization [8]. Based on these developments, researchers have explored various image-processing techniques to optimize PET templates and obtain more reliable CL or SUVR values [8–11]. These pipelines utilize techniques such as masking optimization, precise region of interest delineation, and the determination of appropriate cutoff values, all of which are aimed at reducing errors and enhancing diagnostic accuracy. Consequently, these methods have the potential to become crucial tools for amyloid PET imaging studies.

CapAIBL, an innovative software developed by the Australian eHealth Research Centre, CSIRO (https://milxcloud.csiro.au/)serves as an advanced tool for simplifying and standardizing the quantification of imaging biomarkers without the need for MRI scans [12]. CapAIBL has been used to assess CL in recent studies [13–15]. This software employs multiple PET atlases and a local and dynamic patch-based method to align PET images with gray matter tissue probabilities and cortical surfaces. Using a Bayesian framework, CapAIBL generates consensus PiB estimates for each brain region. The final output provides a cortical surface with an atlas correspondence, where each vertex encodes a raw PiB retention estimation for that location.

In addition to CapAIBL, two other softwares have been developed to calculate the CL values. First, VIZCalc is a novel automated semiquantitative analysis technique without MRI that is primarily used for processing 18F-flutemetamol PET images [10]. Its core feature lies in the weighted average image of the positive and negative template methods, which obtains the optimal template and resolves the issue of overestimation of SUVR values encountered in traditional average template-based standardization. In cases where PET images are judged to be equivocal during visual interpretation, VIZCalc provides effective assistance by utilizing optimal cutoff CL values [10]. VIZCalc can classify equivocal cases into negative and positive categories more accurately. Amyquant is another independent software designed for the semi-quantitative measurement of amyloid PET images [9]. The main objective of this software is to provide a more reliable determination of amyloid positivity. In addition to calculating the global CL value, Amyquant also includes a pipeline to capture local patterns of amyloid accumulation through Z-score mapping. This software supports five currently available amyloid PET tracers: 11C-PiB, 18F-florbetapir, 18F-flutemetamol, 18F-florbetaben, and 18F-NAV4694. The process of using Amyquant requires MRI images and is not fully automatic because the origin of the PET and MRI images around the anterior commissure is manually set.

Despite using these softwares, the consistency of the calculated CL values has not been sufficiently evaluated. Considering the upcoming disease-modifying therapies for Alzheimer’s disease (AD) continuum, objective assessment of beta-amyloid (Aβ) positivity is useful for a precise diagnosis. Therefore, our multi-center study aimed to investigate the agreement of CL values to reduce the cases with an equivocal interpretation of positive or negative Aβ burden. Our goal is to assess their performance and suitability for quantifying the Aβ burden. By examining the agreement between the CL values obtained from these methods, we aimed to determine whether they exhibited consistency or differences among the different analysis pipelines.

## Materials and methods

### Participants

Participants were recruited from three facilities: the National Center for Geriatrics and Gerontology (NCGG) in Ōbu, Aichi (*n* = 109); Kindai University in Osaka-Sayama City, Osaka (*n* = 65); and the Tokyo Metropolitan Institute for Geriatrics and Gerontology (TMIG) in Tokyo (*n* = 20) (Table 1). They were enrolled in the “Clinical utility of plasma amyloid beta biomarker: a multicenter validation study (CUPAB)” Project (Public Database Registration number: jRCTs032200043, Initial CRB approval date: March 25, 2020), approved by the Certified Review Boad of National Institutes for Quantum Science and Technology (CRB3180004), and all participants provided informed consent. The study adhered to the principles outlined in the Declaration of Helsinki and followed the “Ethical Guidelines for Medical and Health Research Involving Human Subjects” issued by the Ministry of Health, Labour, and Welfare in Japan.

**Table 1:** The demographic data of the participants and the name of the device at each facility

	NCGG	Kindai University	TMIG
Total (male/female)	107(53/54)	65(32/33)	20(7/13)
Age at Time of Scan (range)	70.1 ± 9.2 (50–87)	72.5 ± 7.9 (56–85)	75.4 ± 3.0 (71–82)
Clinical diagnoses (AD/MCI/nonAD/CN)	32/25/5/45	23/30/8/4	0/10/0/10
Combinations of PET scanner	Biograph 16 True Point and GE Discovery IQ.x	Discovery PET/CT710	Discovery PET/CT 710 GE Discovery MI

Data are shown as absolute numbers or the mean ± standard deviation

*AD* Alzheimer’s disease; *CN* cognitively normal; *MCI* mild cognitive impairment; *nonAD* non-AD dementia; *NCGG* National Center for Geriatrics and Gerontology; *TMIG* Tokyo Metropolitan Institute for Geriatrics and Gerontology

All the participants underwent a battery of neuropsychological tests. Based on clinical assessments by outpatient physicians, participants were clinically categorized as having AD, mild cognitive impairment (MCI), non-AD dementia (nonAD), or cognitively normal (CN). Based on the medical records, laboratory tests, neuropsychological tests, and MRI, participants with any pertinent medical, neurologic, or psychiatric diseases such as cerebrovascular diseases, brain tumors, and mood disorders other than AD and MCI were excluded from the selection.

### Imaging protocol

All enrolled participants were injected with18F-flutemetamol and PET scans were performed at their respective facilities in accordance with the study protocol. NCGG imaging was performed using two PET-CT cameras (Biograph 16 True Point; Siemens Healthcare, Erlangen, Germany and GE Discovery IQ.x; GE Healthcare, Japan). Imaging was performed at Kindai University using Discovery PET/CT710 (GE Healthcare). The TMIG employed two devices: Discovery PET/CT710 and Discovery MI. Although different facilities employed distinct PET equipment, both devices followed the same imaging protocol, capturing images over a 20-min period starting 90 min after intravenous injection, with four frames each lasting 300 s. Data from the averaged images of all four frames with motion correction were utilized for the CL calculation. All three facilities were certified as imaging facilities for amyloid PET by the Japanese Society of Nuclear Medicine. All PET scans were performed under good clinical practice (GCP) according to the standard imaging protocols (ver.2-1) defined by the Japanese Society of Nuclear Medicine (http://jsnm.org/archives/3561/).

For analyses using Amyquant, three-dimensional T1-weighted images were scanned using MRI scanners from different vendors, as described later. MRI examinations were conducted using Philips MRI scanners: Achieva 3.0 T, Achieva dStream 1.5 T, and Ingenia Prodiva 1.5 T; GE Healthcare MRI scanners: SIGNA Explorer 1.5 T and Signa HDxt 1.5 T; and Siemens MRI scanners: Skyra 3 T and MAGNETOM Vida 3 T.

### Centiloid measurement

**Fig. 1:**
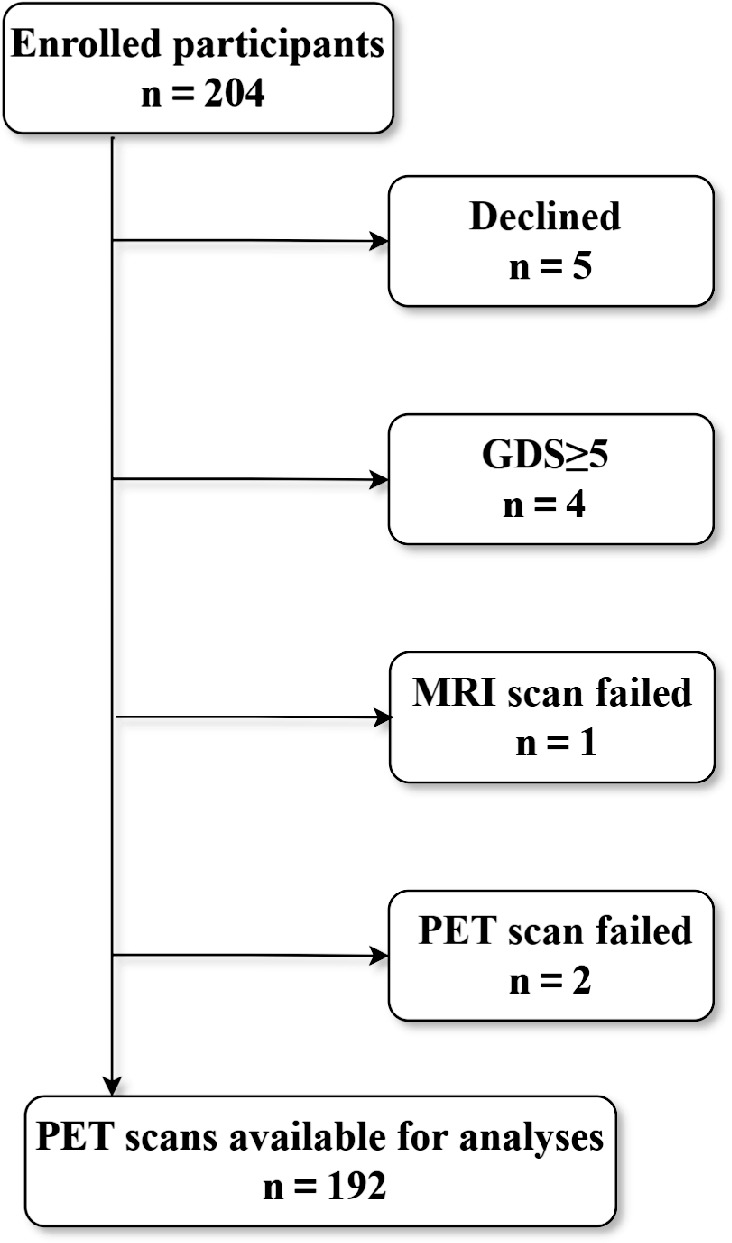
Flowchart showing participant selection for the study. Of the 205 participants enrolled in the BATON project since July 2020, PET scans were performed for 196 participants. Following exclusion of one participant who did not meet the GDS criteria and three with MRI deficiencies, the final analysis was performed on a dataset of 192 participants

Preprocessed data from all three facilities were collected and sent to the NCGG. At NCGG, each individual's data were processed separately using three different pipelines: the CapAIBL, VIZCalc, and Amyquant methods, to calculate their respective CL values [9, 10, 12]. These three methods follow predefined pipeline procedures, which include initial steps such as anatomical standardization, co-registration, localization, and template utilization. Notably, in all three methods, the calculation of CL was based on SUVR measurements, with the whole cerebellum (WC) serving as the reference region. The SUVR was converted to CL using specific formulas defined by each software package. The distribution of participant counts with different clinical diagnoses across various CL ranges is shown in Fig. 1.

### Statistical analysis

All statistical analyses were performed using the standard software (Stata 16.0; Stata Corp., College Station, TX, USA). Spearman's rank correlation coefficient was used to assess the degree of correlation between the CL values obtained from the three different methods, with the aim of evaluating the strength of the relationships among these values. Linear regression was employed to investigate the linear relationships between the CL values obtained using different methods, providing insights into their consistency. Friedman tests were conducted to determine whether there were significant differences in the CL values among the three distinct methods, with the objective of identifying variations in the CL values, even if they exhibited high correlations. Post-hoc Wilcoxon signed-rank tests were used to uncover specific differences between method pairs because the CL data did not follow a normal distribution. Significance was set at Bonferroni-corrected *p* < 0.05. Additionally, a Bland–Altman analysis was performed to detect and assess any potential systematic bias that might exist across the three distinct methods, with the aim of evaluating the agreement and consistency in measurements between the methods, especially by identifying any patterns of bias or outliers in their differences.

## Results

Of the 205 participants enrolled in the CUPAB project since July 2020, PET scans were performed on 196 participants. However, during the analysis, one participant who did not meet the Geriatric Depression Scale criteria was excluded, and three participants were excluded because of the non-availability of MRI. The final dataset for analysis comprised 192 participants (Fig. 1), who were categorized as AD (*n* = 55), MCI (*n* = 65), nonAD (*n* = 13), and CN (*n* = 59). Table 1 summarizes the demographic data of the participants and the name of the device at each facility.

### Agreement correlation analysis

**Fig. 2:**
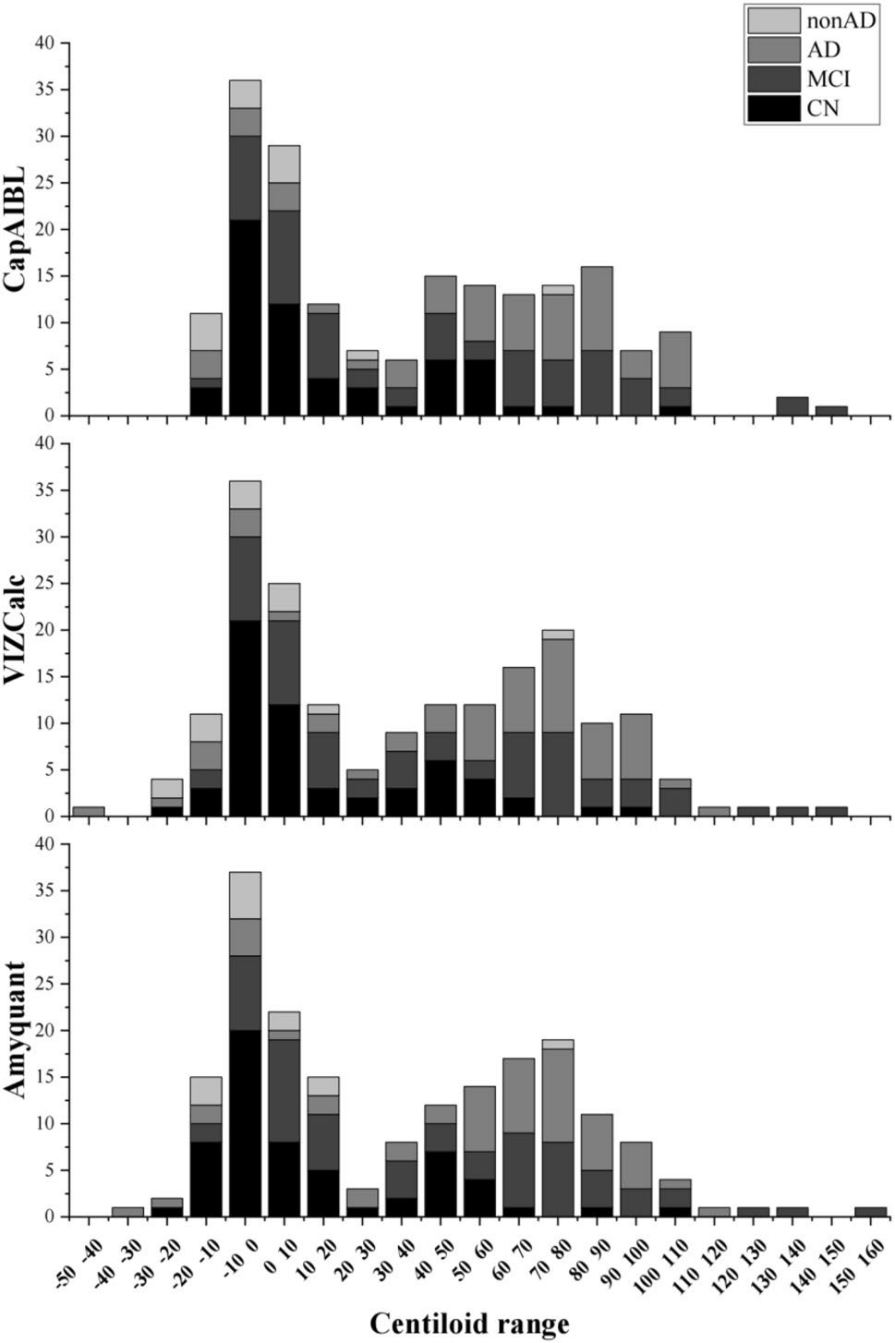
Distribution of participant counts across software by CL value ranges, categorized by different clinical diagnoses. The distribution of the number of participants with different clinical diagnoses along with CL values is shown in Fig. 2. The horizontal axis represents the Centiloid ranges, and the vertical axis represents the number of participants

**Fig. 3:**
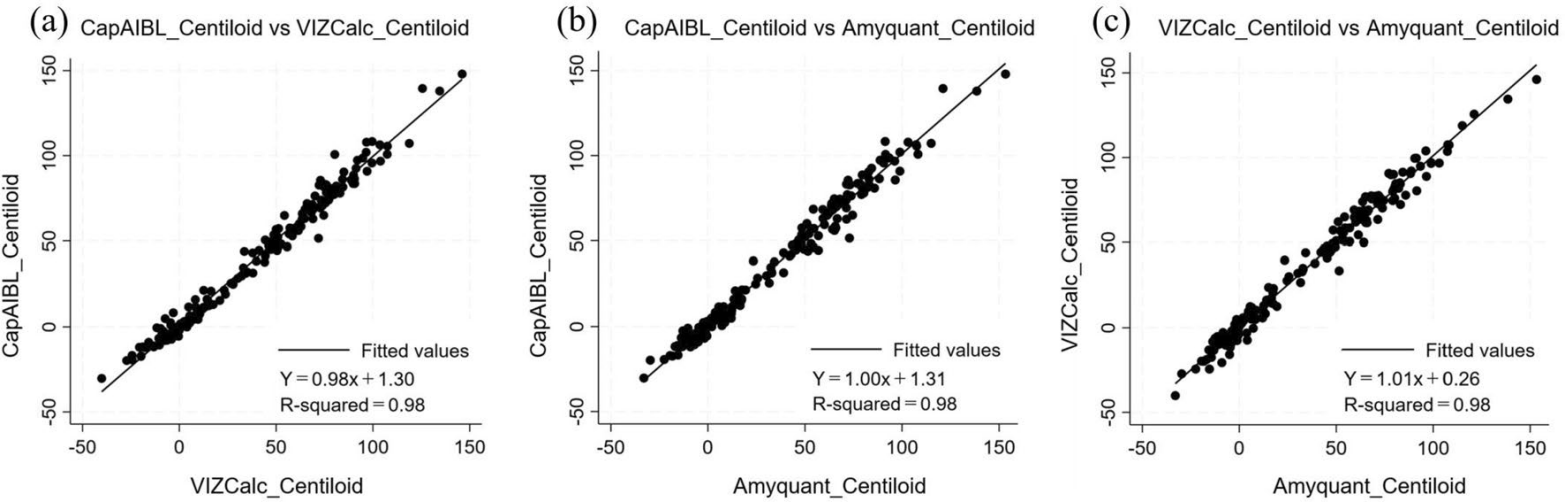
Scatter plots of Centiloid values between two different methods. **a** Scatter plot between the CapAIBL and VIZCalc methods. **b** Scatter plot between the CapAIBL and Amyquant methods. **c** Scatter plot between the VIZCalc and Amyquant methods. The three graphs depict the pairwise linear regression analyses involving CapAIBL, VIZCalc, and Amyquant. The linear relationships were examined for each combination. Notably, the resulting regression plots for all pairs of variables display strikingly similar patterns, with the fitted regression lines closely coincident. This consistency suggests a robust linear relationship between pairs of variables. Furthermore, the slopes and intercepts of the linear regression equations remained within reasonable ranges, which is consistent with the validation criteria defined by Klunk et al. in 2015 [1]

Table 2 shows the correlations between the three methods (CapAIBL, VIZCalc, and AmyQuant) for the calculation of CL values. Significant positive correlations were observed between CL values derived from CapAIBL and VIZCalc (rho = 0.99, *p* < 0.001), CapAIBL and Amyquant (rho = 0.99, *p* < 0.001), and VIZCalc and Amyquant (rho = 0.99, *p* < 0.001). The distribution of participant counts with different clinical diagnoses across various CL ranges is shown in Fig. 2. Figure 3 shows the scatter plots and regression lines that evaluate the correlation between different methods regarding CL values and examine the potential presence of systematic bias between these methods. The slopes, intercepts, and R-squared correlation coefficients between the two different methods (CapAIBL vs. VIZCalc, CapAIBL vs. Amyquant, and VIZCalc vs. Amyquant) were 0.98, 1.30, 0.98, 1.00, 1.31, 0.98, and 1.01, 0.26, 0.98, respectively. These results were consistent with the criteria for validation, suggesting a slope between 0.98 and 1.02, intercept within the range of − 2 to + 2 CL, and an R2 correlation coefficient of 0.98 [1].

**Table 2:** Correlation analysis of Centiloid values between two different methods

	ρ	*p*-value
CapAIBL vs. VIZCalc	0.99	< .001*
CapAIBL vs. Amyquant	0.99	< .001*
VIZCalc vs. Amyquant	0.99	< .001*

^*^Spearman Correlation analysis revealed no significant differences among the pairwise comparisons of the three methods

Despite the high correlation in CL values among the three methods, there was a significant difference in the Friedman test (*p* < 0.001) (Table 3). To assess the differences between each pair of methods, post-hoc Wilcoxon signed-rank tests were employed because of the non-normal distribution of the data. As a result, a significant difference was solely observed between CL values from CapAIBL and Amyquant (36.1 ± 39.7 vs. 34.9 ± 39.4; *p* < 0.001). In contrast, no significant differences were observed between CapAIBL and VIZCalc (36.1 ± 39.7 vs. 35.5 ± 40.1; *p* = 0.81), and between VIZCalc and Amyquant (35.5 ± 40.1 vs. 34.9 ± 39.4; *p* = 0.18), respectively.

**Table 3:** Comparison of Centiloid values among three methods

	CapAIBL	VIZCalc	Amyquant	*p*-value
Centiloid value	36.1 ± 39.7**	35.5 ± 40.1	34.9 ± 39.4	< 0.001*

Data are shown as the mean ± standard deviation

^*^For the comparison between CapAIBL, VIZCalc and Amyquant (Friedman test p < . 001)

^**^For the comparison between the CapAIBL and Amyquant (Bonferroni-corrected p < . 001)

**Fig. 4:**
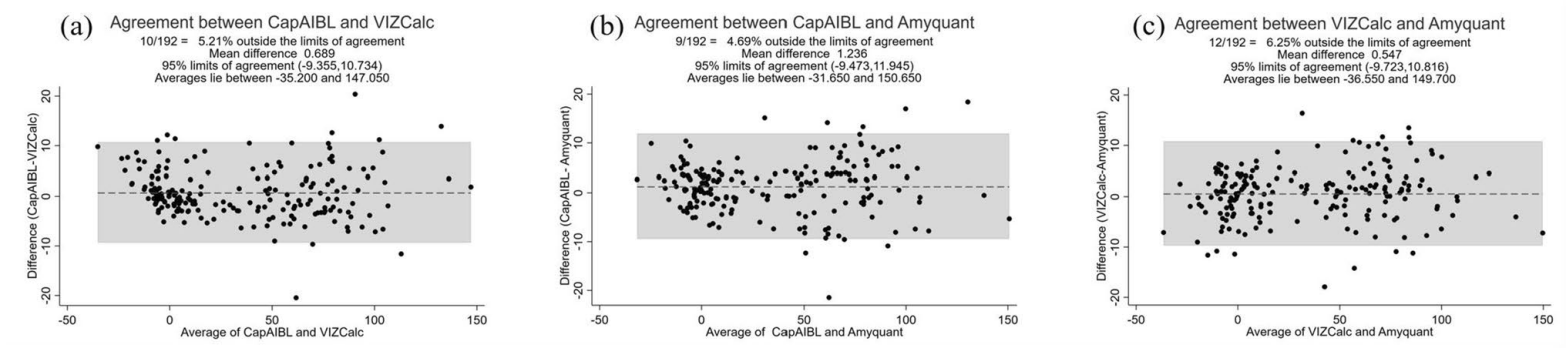
Bland–Altman plots showing agreements of Centiloid values between two different methods. **a** Bland–Altman plot between the CapAIBL and VIZCalc methods. **b** Bland–Altman plot between the CapAIBL and Amyquant methods. **c** Scatter plot between the VIZCalc and Amyquant methods. The Bland–Altman analysis, which is visually intuitive, revealed no apparent systematic bias among the three methods

Finally, Bland–Altman plots were used to visually gauge the concordance between the two measurement methods. Figure 4 depicts Bland–Altman plots comparing the CL values between pairs of methods. In each analysis, approximately 94–95% of the participants were within the limits of agreement. Importantly, systematic errors in all comparisons were negligible (i.e., 0.59, 0.62, and 1.21, respectively) (Fig. 4).

## Discussion

This study assesses the consistency of Centiloid values obtained by three methods, CapAIBL, VIZCalc, and Amyquant, for 18F-flutemetamol PET. A novel aspect of this study was the evaluation of the number of participants recruited from three different facilities using different PET scanners. Using various agreement correlation analyses, a consistent relationship in the calculations of CL values among the three different methods—CapAIBL, VIZCalc, and Amyquant, for semiquantifying 18F-flutemetamol amyloid PET images, even across different facilities using different PET scanners, was clarified. Furthermore, the results of the linear regression adhered to the criteria established by Klunk et al. in 2015 [1]. The adherence to these criteria underscores the robustness and reliability of the analysis. Analysis assessing the consistency of CL values among these methods holds considerable importance, as it demonstrates strong agreement, highlighting their reliability and interchangeability for amyloid quantification using the CL scale.

The Food and Drug Administration mandates that amyloid PET images be qualified using a binary system of positive or negative scans [16]. Despite the relatively high interrater agreement of visual assessment in various amyloid PET tracers (i.e., K = 0.8–0.9), approximately 10% of cases were interpreted as equivocal between positivity and negativity [17–19]. Considering the benefits of early intervention using disease-modifying therapies, early screening using more objective methods to interpret amyloid PET findings is crucial. There have been reports that semi-quantitative analysis using the SUVR is useful for the interpretation of such equivocal cases [18–20]. Quantitative evaluation, including the SUVR of amyloid PET, is vulnerable to variations arising from differences in tracers, timing of imaging acquisition, PET scanners, and imaging protocols [21]. Additionally, the major shortcoming of SUVR is its inability to provide a unified evaluation for different radiotracers and target/reference region settings.

On the other hand, the CL scale represents a substantial advancement in amyloid imaging and offers standardized units that address several key challenges associated with standardized SUVR measurements. Advantages of CL include enhanced data comparability across different sites and tracers, consistent quantification, cutoffs for improved tracking of longitudinal changes, and simplified data interpretation [1]. Therefore, validating the consistency and reliability of the CL calculations across different pipelines is crucial.

The remarkable consistency demonstrated by the three CL calculation methods used in this study is particularly intriguing. In particular, when conducting Bland–Altman analysis, which entailed pairwise comparisons among the three groups, the most notable average difference observed was a mere 1.2 units between CapAIBL and Amyquant. Despite concerns raised in various studies about factors such as differences in template utilization, standardization methods, and technical intricacies that could potentially affect the SUVR results preceding CL value calculations, our findings remain significant [22, 23]. In recent years, considerable progress has been made in calculating CL values and/or SUVR from amyloid PET images. In the SUVR measurements, the WC was chosen as the reference region over the pons because of its higher stability and sensitivity. Cho SH et al. stated that using the WC as a reference region resulted in the smallest variance in the centiloid scale of flutemetamol [24]. Klunk et al. also found the WC was the reference region that gives the smallest standard deviation in calibrating centiloid scale values [1]. Therefore, the WC is preferred as a reference region in this context. If other regions were selected for the reference region, the variance in the centiloid scale values would be larger than those in the present study, although the consistency across the calculation software may be reserved [25].

Differences in the final results owing to the varied data processing techniques among the three pipelines were anticipated. Both CapAIBL and VIZCalc utilize non-MRI methods; however, they differ in the creation of reference image sets. CapAIBL uses an adaptive template that is a linear combination of an Aβ negative and Aβ positive template, with a weight optimized by maximizing the normalized mutual information between the adaptive template and the affine-registrated individual image. After optimization, spatial normalization is performed directly on the template without the need for an MRI [13]. On the other hand, VIZCalc uses zero-mean normalized cross-correlation to compute the similarity of each candidate template with the participant's PET image and selects the candidate template with the highest similarity as the optimal template. The individual PET image is spatially normalized to the individually selected template image with a program package that includes a method to express a deformation field using basis functions [10]. SUVR may be overestimated when using standardized PET images with an average template compared to standardized MR images [14, 26]. An optimal template, which is a weighted average image of positive and negative templates that maximizes the similarity to the participant's PET image, can reduce the standardization error for each patient [27]. In contrast to Amyquant, VIZCalc and CapAIBL use PET templates as intermediaries for PET image standardization and directly calculate CL values from PET images. The variations noted in the Wilcoxon signed-rank tests between Amyquant and the other two methods may be primarily attributed to differences in the anatomical standardization pipeline. However, it is important to emphasize that the differences among the three methods were minimal, indicating their limited diagnostic significance.

The conversion formulae from SUVR of flutemetamol to CL values in each software are as follows, CapAIBL: CL = 112.74 × SUVR—121.53; VIZcal: CL = 122.83 × SUVR—126.13; and Amyquant: CL = 121.42 × SUVR—121.16. The formulae are mostly similar and only have slight differences. However, it is difficult to infer the cause of the difference in CL values from directly comparing the formulas. This is because the SUVR values calculated by the programs are already expected to be different depending on the processing pipelines. In fact, the similarity seen from the calculation formula does not seem to correspond to the results of the correlations and the Bland–Altman plot across the softwares.

This study had several limitations. First, the inclusion of samples as a continuum resulted in an uneven distribution of CL values. In the Bland–Altman plots, it is evident that the average CL values are densely distributed in the low and high ranges, particularly in the lower range. However, the distribution in the intermediate equivocal range was relatively sparse. An uneven distribution of the CL values can lead to inaccurate consistency assessments. This might cause an overestimation or underestimation of agreement across the entire CL range, owing to significant discrepancies in certain ranges. Second, this study exclusively confirmed the effectiveness of 18F-flutemetamol and lacked evidence of consistent results with other radiotracers. Third, this study only conducted consistency validation for CL calculations using WC as the reference region. Performance validation of the three pipelines for CL measurements using other reference regions such as the pons and WC plus the brainstem was not conducted. Lastly, this study examined the consistency of CL of flutemetamol PET across calculation softwares, not across PET facilities (such as PET cameras). If the CL differences between PET camera models are to be examined, it would be necessary to scan the same individual with different PET cameras. This is an important research topic that should be explored in the future.

In conclusion, this groundbreaking study validated the consistent performance of the CapAIBL, VIZCalc, and Amyquant methods in semiquantifying 18F-flutemetamol amyloid PET images across diverse facilities with varying scanners. This analysis unequivocally underscores the substantial agreement between the CL values derived from these methods, thereby emphasizing their robust reliability and practical interchangeability for amyloid quantification on the CL scale. Despite the marginal differences in CL measurements when comparing Amyquant to other methods, we posit that these variances have minimal potential to affect the interpretation of amyloid-related findings.

## Data Availability

The datasets generated and analyzed during the current study are available from the corresponding author on reasonable request.
